# Pre-phase strategy to mitigate first cycle effect in diffuse large B cell lymphoma

**DOI:** 10.1186/s43046-022-00116-5

**Published:** 2022-05-09

**Authors:** A. H. Rudresha, Syed Adil Hassan, A. Sreevalli, D. Lokanatha, M. C. Suresh Babu, K. N. Lokesh, L. K. Rajeev, Smitha Saldanha, Antony G. F. Thottian, Kanika Sharma, Linu Abraham Jacob

**Affiliations:** Department of Medical Oncology, Kidwai Memorial Institute of Oncology, Dr. M H Marigowda Rd., Hombegowda Nagar, Bengaluru, Karnataka 560029 India

**Keywords:** Pre-phase, First cycle effect, DLBCL

## Abstract

**Context:**

Treatment-related toxicities in DLBCL (diffuse large B cell lymphoma) patients are higher in the initial phase of treatment (first cycle effect). Implementation of pre-phase treatment before definitive chemotherapy had been shown to alleviate some of these side-effects in a non-randomized study conducted earlier in our institute (Lakshmaiah et. al., Eur J Haematol 100:644-8, 2018).

**Aims:**

This study was aimed at validating the role of pre-phase treatment in newly diagnosed DLBCL patients.

**Settings and design:**

All newly diagnosed patients with DLBCL above the age of 18 years were evaluated for eligibility and prospectively enrolled. A single-arm prospective study was conducted at the Department of Medical Oncology, in our institute from July 2015 to December 2019.

**Methods and material:**

Patients received vincristine and prednisolone as pre-phase treatment for 7 days after which definitive chemotherapy was instituted on day 1. They were followed up for 30 days post-first cycle chemotherapy.

**Statistical analysis used:**

Paired Student’s *t* tests and Wilcoxon signed-ranks test were used for comparison of various clinical variables as appropriate. *P* value of less than 0.05 was considered significant.

**Results:**

Among the 180 patients who were included in study, performance status improvement was noted in significant number of patients (*p* < 0.001). 38.4% achieved an ECOG (Eastern Cooperative Oncology Group) performance status of 0 post-pre-phase therapy. Febrile neutropenia was observed in 12.8% in the present cohort as compared to the historical non-pre-phase cohort (34%).

**Conclusions:**

Pre-phase therapy significantly improves the performance status and diminishes neutropenia rates in DLBCL patients.

## Key messages

Pre-phase therapy is a simple and cost-effective method to improve tolerance to definitive therapy. It enhances the performance status and decreases rates of febrile neutropenia in patients with DLBCL. We suggest using pre-phase in all elderly patients of DLBCL as well as those with a decreased performance status.

## Background

DLBCL (diffuse large B cell lymphoma) constitutes around one-third of all the cases of non-Hodgkin's lymphoma (NHL) [1]. Treatment-related side-effects including longest duration of neutropenia, deepest neutrophil nadir, and highest rate of treatment-related mortality are observed post-first cycle of chemotherapy (first-cycle effect) [2]. “Pre-phase” treatment consisting of a low dose chemotherapy for 5 to 7 days prior to definitive chemotherapy is recommended in elderly DLBCL patients to improve the performance status and to ameliorate these toxicities [3]. After a prospective non-randomized study conducted in our institute yielded encouraging results [4], this study was aimed at further validating the role of pre-phase treatment in newly diagnosed DLBCL patients. The primary objective of the study was to assess the benefits of pre-phase treatment in terms of improvement in ECOG performance status, nadir ANC (absolute neutrophil count), the incidence of febrile neutropenia and mortality in the initial 30 days post-first cycle of CHOP chemotherapy.

## Methods

Newly diagnosed patients with DLBCL above the age of 18 years were prospectively enrolled into this study after obtaining written informed consent. The study was conducted at the Medical Oncology Department in our tertiary care center from July 2015 to December 2019 after obtaining institutional ethics committee approval. Excision biopsy of the lymph node was the preferred modality for diagnosis confirmation, in absence of which, a core needle biopsy of involved extra-nodal organ or lymph node was performed. Histopathological examination followed by IHC (immunohistochemistry) markers (LCA, CD20, PAX 5, CD3, CD10, BCL2, BCL6, MUM1, MYC, cyclin D1) were used for confirming the diagnosis of DLBCL and for dividing into germinal center B cell type (GCB) and non-GCB (NGCB) subtypes as per the Hans algorithm. The staging workup included a contrast-enhanced computed tomography (CECT scan) with a bone marrow biopsy from the unilateral iliac crest or positron emission tomography (PET/CT). Patients were staged as per the Cotswolds modification of the Ann Arbor staging system [3].

Primary CNS (central nervous system) lymphoma, transformed DLBCL, relapsed cases, and HIV (human immunodeficiency virus)-associated DLBCL were excluded. Patients below 60 years with an ECOG performance status of 0 were not included, they were treated with R ± CHOP-21 (rituximab ± cyclophosphamide, hydroxydaunorubicin, oncovin, prednisone) upfront. Written informed consent was obtained from all the participants. After fulfillment of eligibility criteria, pre-phase therapy was administered for 1 week, which was followed by the definitive chemotherapy.

Pre-phase treatment consisted of 1 mg fixed dose of vincristine on − 6th day as intravenous push and 7 days of prednisolone (100 mg orally, from day − 6th to day 0). All patients received definitive chemotherapy (CHOP-21 with or without rituximab) on day 1 as per standard protocol. Rituximab could not be administered for all patients in view of financial constraints in our setup. The chemotherapy cycles were repeated at an interval of 21 days. Prophylactic growth factor (G-CSF) was used in patients with age more than 60 years and in patients with multiple comorbidities as per physician’s discretion. Patients who developed febrile neutropenia received G-CSF in the subsequent cycles of chemotherapy. Supportive treatment as per the institute protocol was followed.

## Results

### Patient demographics and clinical characteristics

Hundred and eighty-eight new patients with DLBCL were found eligible for the study. The median age of the patients was 56 years (range 18–83 years). There was a male preponderance (58.5%). Over half of study population had extra-nodal involvement along with nodal disease. Hundred and thirteen patients (60.1%) had advanced disease (stage III or IV). Higher proportion of population were categorized into the NGCB subtype based on the Hans algorithm. Prophylactic G-CSF was given to 40.4% (*n* = 76) patients in the pre-phase cohort. Rituximab-based therapy was administered in 56.4% of patients (Table 1).

**Table 1:** Clinical profile of all patients receiving pre-phase therapy in comparison to the historical pre-phase cohort

Clinical variables	Historical pre-phase cohort (***N*** = 50)	Total pre-phase cohort (***N*** = 188)
**Mean age (years)**	51.7	52.9
**Male**	29 (58%)	110 (58.5%)
**Female**	21 (42%)	78 (41.5%)
**Mean LDH (U/L)**		
**Prior to pre-phase**	441.4	398
**Post-pre-phase**	338.6	301
**Cell of origin**		
**GCB**	21 (42%)	85 (45.2%)
**Non-GCB**	29 (58%)	103 (54.8%)
**Stage (*N*)**		
**I**	0 (0%)	15 (8%)
**II**	17 (34%)	60 (31.9%)
**III**	13 (26%)	39 (20.7%)
**IV**	20 (40%)	74 (39.4%)
**Extra-nodal disease**	29 (58%)	100 (53.2%)
**IPI score (score)**		
**Low risk (0, 1)**	9 (18%)	58 (30.9%)
**Low intermediate (2)**	19 (38%)	58 (30.9%)
**High intermediate (3)**	12 (24%)	41 (21.8%)
**High risk (4, 5)**	10 (20%)	31 (16.5%)
**Treatment**		
**Rituximab based**	16 (32%)	106 (56.4%)
**Non Rituximab based**	34 (68%)	82 (43.6%)
**Prophylactic growth factor (G-CSF)**	12 (24%)	76 (40.4%)

### Impact of pre-phase

**Fig. 1:**
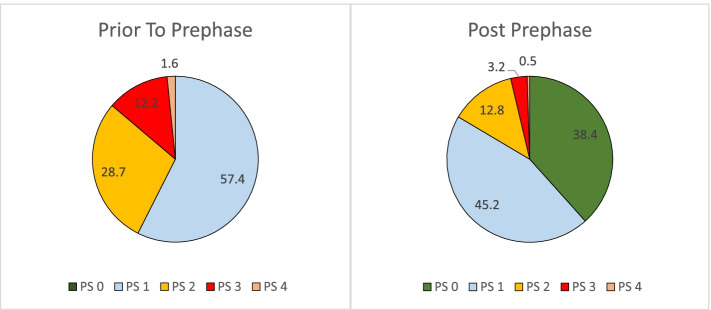
Comparison of ECOG performance status of patients prior to and post-pre-phase therapy

**Fig. 2:**
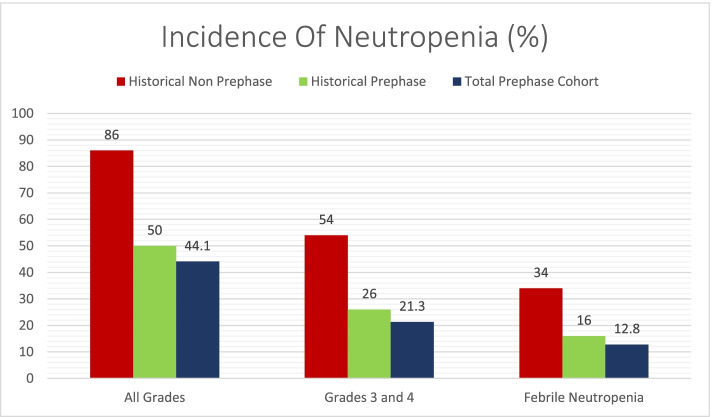
Incidence of neutropenia on day 10 of 1st cycle chemotherapy. In comparison to historical cohort of patients (1)

Improvement in performance status was observed in a significant number of patients after pre-phase treatment (*p* < 0.001, Wilcoxon signed-rank test) (Fig. 1). Among the study population 83.6% achieved an ECOG performance status of either 0 or 1 before starting definitive chemotherapy compared to 57.4% before staring pre-phase treatment. Significant fall was seen in mean serum LDH (lactate dehydrogenase) levels post-pre-phase therapy indicating decrease in tumor burden (*p* < 0.001, paired samples *t* test). Neutropenia on D10 post-first cycle of definitive chemotherapy was noted in 44.1%. Febrile neutropenia was observed in 12.8%. Figure 2 shows the rates of neutropenia and febrile neutropenia noted in the current report in comparison with the prior report published from our institute on the effect of pre-phase. Early mortality defined as death within 30 days of diagnosis was noted in 3.7% of patients.

## Discussion

Pre-phase therapy was first proposed by Pfreundschuh M et al. in 2004 with the objective of reducing “first-cycle effects” in elderly patients with aggressive lymphomas. However, since the pre-phase treatment application was not regularly documented, statistical quantification of this clinical experience was not published [2]. Drugs used for pre-phase and the dosage employed has seen some variation over the years. Initial trials from Germany have used a combination of Vincristine and Prednisolone for 7 days. A Chinese study which evaluated its role in gastric DLBCL used vincristine 1 mg and cyclophosphamide 200 mg [5]. Owens et al. from MSKCC (Memorial Sloan Kettering Cancer Centre) employed a pre-phase consisting of prednisolone (50–100 mg) for 5–10 days and rituximab 375 mg/m^2^ × 1 day completed 14 days prior to R-CHOP-21 as part of a larger geriatric assessment validation study in NHL [6]. A prospective phase II study (LNH09-7B) used pre-phase in elderly in combination with ofatumumab based therapy.

After introduction of the pre-phase treatment, trials have noted a drop in the number of treatment-related deaths and improvement in performance status [3, 7]. In addition to these effects, Cui et al also demonstrated an improvement in the 5-year progression-free survival (PFS) rate (63% vs. 31%, *p <* 0.021) in comparison to patients who received conventional-dose CT alone [5]. Owens et al. documented an improvement in Karnofsky performance status from a median of 70 to 80% post-pre-phase treatment [6]. In our study, we have noted a significant improvement in PS after pre-phase since 83.6% achieved ECOG performance status 0 or 1 post-pre-phase compared to 57.4% prior to pre-phase treatment.

Grade 3 or 4 neutropenia is considered the most common severe side-effect of chemotherapy and infection-associated neutropenia results in most treatment-related mortality. A Korean study assessing elderly DLBCL reported neutropenia in 63.0% of patients, majority (> 90%) of whom had grade 3 or higher toxicity. More than three-fourths of neutropenia cases (78.4%) developed after the first cycle [8]. RICOVER-60 trial reported ≥ grade 3 neutropenia in around 61% among its different study cohorts. This is in contrast to the LNH09-7B study where grade 3–4 neutropenia was observed in 20.8% of the patients. These studies focused on elderly DLBCL. A previous report from our institute indicated that febrile neutropenia rates were high in our set up (34%). Therefore, in an effort to reduce these rates without compromising the dose intensity of drugs, all patients above the age of 18 years were included in this study. The preliminary report found a drastic decrease in febrile neutropenia rates to 16% with pre-phase. In this cohort, we report a further decrease in our febrile neutropenia rates to 12.8%. This may be attributable to the liberal use of growth factor support.

Our study has certain limitations. This is a single-institute study. The effect on survival has not been reported due to short follow-up. The cohort of patients who benefit maximum from pre-phase is yet to be identified. Though previously published literature suggest the maximum benefit to be in elderly and those with PS 2 or greater prior to therapy, in our cohort, patients of all ages and PS benefited from pre-phase therapy.

## Conclusions

The present study has further strengthened the role of pre-phase treatment prior to definitive chemotherapy (CHOP ± rituximab) in terms of improvement of the performance status and decreasing rates of neutropenia and febrile neutropenia. We suggest using pre-phase in all elderly patients of DLBCL as well as those with a decreased performance status.

## Data Availability

Not applicable.

## Abbreviations

DLBCL: Diffuse large B cell lymphoma
ECOG: Eastern Cooperative Oncology Group
NHL: Non-Hodgkin’s lymphoma
IHC: Immunohistochemistry
GCB: Germinal center B cell type
NGCB: Non-germinal center B cell type
CECT: Contrast-enhanced computed tomography
PET/CT: Positron emission tomography/computed tomography
CNS: Central nervous system
HIV: Human immunodeficiency virus
R ± CHOP-21: Rituximab ± cyclophosphamide, hydroxydaunorubicin, oncovin, prednisone
G-CSF: Granulocyte colony stimulating factor
ANC: Absolute neutrophil count
LDH: Lactate dehydrogenase
MSKCC: Memorial Sloan Kettering Cancer Centre
PFS: Progression-free survival
IPI score: International Prognostic Index score
